# Advances in the synthesis and applications of porous carbon materials

**DOI:** 10.3389/fchem.2023.1205280

**Published:** 2023-07-11

**Authors:** Mei Ni, Lei Zhou, Yancen Liu, Runtao Ni

**Affiliations:** ^1^ Department of Basic Courses, China Fire and Rescue Institute, Beijing, China; ^2^ Yangtze Delta Region Institute, University of Electronic Sciences and Technology of China, Huzhou, China; ^3^ Institute of Fundamental and Frontiers Sciences, University of Electronic Sciences and Technology of China, Chengdu, China; ^4^ Administration for Market Regulation of Zhengding, Shijiazhuang, China

**Keywords:** porous carbon materials, synthesis, adsorption, energy storage, catalysis

## 1 Introduction

The progress of human civilization depends on the development of all kinds of materials. The establishment of modern science has led to the rapid development of synthetic materials. However, increasing energy demand and environmental pollution urgently require the search for new materials to solve the energy and environmental crisis.

Carbon is an extremely abundant element in nature and provides the basis for all life on Earth (Li et al., 2008; Toth et al., 2016). The carbon atom has six electrons outside the nucleus, and its outermost electron arrangement is 2*s* ^2^2*p* ^2^, which shows a strong ability to form covalent bonds (Krueger, 2010). Porous carbon materials have advantages such as chemical stability, low density, high thermal conductivity, high electrical conductivity, and high mechanical strength (Gallo, 2017). Porous carbon materials also have a large specific surface area, adjustable pore size, and functional groups and can be prepared from a wide range of precursors at relatively low cost.

In recent years, a large number of researchers have been devoted to the synthesis and application of porous carbon (Ang, 2019; Liu, 2019; Liu, 2020a; Hwang, 2020; Raj, 2021). Depending on the pore size distribution, the pore structure of carbon materials can be divided into three categories, namely, micropores (pore size <2 nm), mesopores (2 nm < pore size <50 nm), and macropores (pore size >50 nm) (Vu, 2012). The size of the pore structure of porous carbon materials has a significant impact on their performance in practical applications.

**FIGURE 1:**
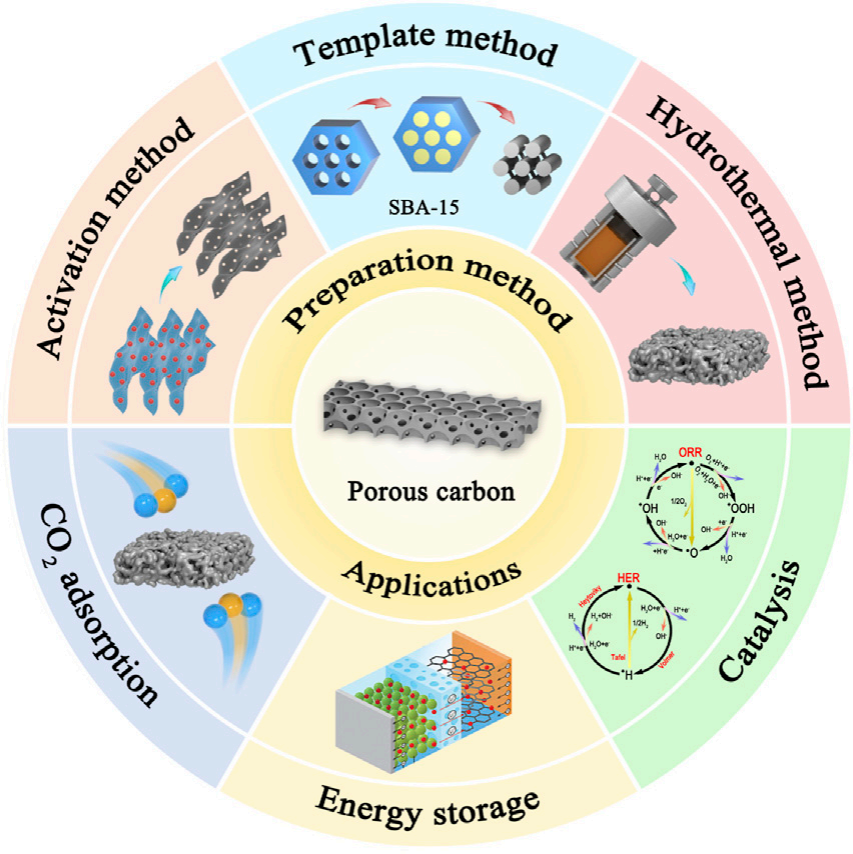
Schematic illustration of the synthesis and application of porous carbon materials.

Due to these advantages, carbon materials are widely used in the fields of adsorption (He, 2019), catalysis (Dong et al., 2020), and energy storage (Peng, 2019). This paper mainly introduces the synthesis and application of carbon materials and describes the main improvement ideas for current carbon materials (Figure 1). Importantly, the future direction of carbon materials is further discussed.

## 2 Synthesis methods of carbon materials

### 2.1 Activation method

Many designs are currently focused on how to increase the specific surface area of carbon materials, which include heat treatment, physical activation, and chemical activation. The physical activation method refers to the use of CO_2_, water vapor, O_2_, and other gases as activation media for the preparation of activated carbon under high-temperature conditions (Sevilla, 2014; Rashidi, 2019; Ma, 2020). The CO_2_ molecules move at a slower rate thermally compared to water vapor activation, resulting in a larger specific surface area and a higher volume of microporous material. However, the small size and low activation of the activating molecules with a single activator result in a large number of blind pores and an ineffective specific surface area of the carbon material (Kim, 2017).

Chemical activation is the reaction of chemical reagents with the carbon source during pyrolysis, commonly known as KOH, NaOH, H_3_PO_4_, and ZnCl_2_ (Molina, 1995; Lillo, 2003). The main factors influencing the preparation of porous carbon materials by chemical activation include the composition of the precursor material, the activation temperature, the activator, and the impregnation ratio (Maia, 2018). A high impregnation ratio (raw material/activator) allows the formation of activated carbon with a high specific surface area, usually between 1:1 and 5:1 (Rashidi, 2016). KOH is the most commonly used activator, and the pore structure of the prepared material is well developed. Prasankumar converted Tasmanian blue gum tree bark into activated carbons through a simple KOH activationand carbonization method. The generated activated carbons have a hierarchically connected mesoporous structure and a surface area of 971 m^2^ g^-1^ with an average pore size of 2.2 nm (Thibeorchews, 2022).

Activated carbon is mainly derived from various organic precursors rich in carbon (bitumen, coal, polymers, etc.). Due to the environmentally unfriendly nature of fossil fuels, a large number of activated carbons prepared from biomass as precursors have attracted a great deal of attention in recent years. They are prepared from natural substances such as walnut shell powder (Pang, 2016; Jiang, 2022), banana stem fibers (Taer, 2021), American poplar fruit (Kumar, 2020), bamboo (Khuong, 2022), castor seed (Neme, 2022), and lotus seed shell (Hu, 2018), synthesizing activated carbons with a high specific surface area.

### 2.2 Template method

Specific surface area and pore size distribution are key factors that affect the properties of carbon materials. The template method is considered to be an effective method for achieving controlled mesoporous structures. The template method can be divided into the hard template method and the soft-template method. The former involves thorough mixing of the hard template with the precursor materials, carbonization, and subsequent removal of the template. The synthesis of porous carbon materials using various structural silica templates, such as SBA-15 (Yan, 2020) and 3D cubic KIT-6 (Karthikeyan, 2022), has been reported in recent years. The pore size and pore volume of the obtained porous carbon materials can be systematically adjusted by changing the size of the template (Sang, 2011). In addition, porous carbon can also be prepared using CaCO_3_, MgO, Mg(OH)_2_, magnesium acetate, magnesium citrate, or magnesium gluconate as templates (Wei, 2019; Zhou, 2020), which can be subsequently removed with dilute hydrochloric acid. Liu found a new solvent-free method for the preparation of mesoporous carbon using mesoporous silica KIT-6 as a hard template for the preparation of mesoporous carbon, overcoming the disadvantages of the conventional filling process (Liu, 2019).

The soft-template method involves the self-assembly and co-condensation of a soft template with a precursor to produce a material with a specific structure (Zhang, 2009). The advantages of the soft-template method are that the template does not require subsequent processing and the experimental steps are simple and environmentally friendly. Soft templates include various triblock copolymers, such as F127 and F108. Peng prepared a unique hierarchical porous N-, O-, and S-enriched carbon foam via a combination of a soft-template method, freeze-drying, and chemical etching (Peng, 2019). The structure offers not only an ultra-high specific surface area but also a network of multiple-scale channels.

### 2.3 Hydrothermal carbonization method

Hydrothermal carbonization is a process in which carbon precursors are gradually hydrolyzed, dehydrated, condensed, and aromatized under high temperature and pressure using water as a solvent and eventually converted into carbon materials. This method is milder and allows for autonomous control of product morphology and better regulation of pore size distribution. In the hydrothermal carbonization process, there are many factors that influence the properties of the carbon material, such as the hydrothermal temperature, the rate of temperature increase, and the holding time. The specific surface area of the carbon material produced is generally low, and the pores are not well developed, so it is often used in combination with activation to obtain porous carbon materials with a high specific surface area.

Liu prepared nitrogen-doped porous carbon materials with a specific surface area of up to 2,864 m^2^ g^-1^ and a total pore volume of 1.6 cm^3^ g^-1^ by hydrothermal treatment of biomass raw materials and the addition of an activator, KOH, to the aqueous solution, followed by high-temperature pyrolysis and activation (Liu, 2016). Veltri prepared a nitrogen–oxygen co-doped biomass-based carbon material by hydrothermal charring of orange juice with a specific surface area of 1,725 m^2^ g^-1^ (Veltri, 2020). The pore structure tended to be reasonable, and the mass fractions of nitrogen and oxygen were as high as 5.65% and 5.38%

## 3 Applications

### 3.1 Application of porous carbons in adsorption

The International Panel on Climate Change (IPCC) report shows that the atmospheric concentrations of greenhouse gases (mainly CO_2_) continue to increase (Lunn, 2021). Porous carbon has a wide range of sources, stable physical and chemical properties, and fast adsorption and desorption rates. It is a kind of CO_2_ adsorption material with great potential for application.

The carbonized coconut shell was modified with urea at 350°C and activated with K_2_CO_3_ to produce a nitrogen-doped carbon material with good CO_2_ adsorption properties (Yue, 2018). He used polydopamine and melamine as carbon sources and CaCO_3_ nanoparticles as templating agents to synthesize porous carbon materials, which showed high CO_2_ adsorption capacity and selectivity at room temperature (He, 2019). Pluronic P123 as a soft template was polymerized with D-glucose by the hydrothermal method and activated by CO_2_ to produce a porous adsorbent with high microporous content (Nicolae, 2020). The CO_2_/N_2_ adsorption selectivity of 9 was achieved at 6.00 mmol g^-1^.

### 3.2 Application of porous carbon in energy storage

In order to mitigate climate change and environmental pollution caused by excessive use of fossil energy, clean and sustainable alternative energy sources are urgently needed around the world (Weigelt, 2016; Azcárate, 2017). In the past decades, a large number of researchers have been devoted to the development of new types of energy storage (Gondal, 2017; McKone, 2017). Carbon is widely used in energy storage and has the advantages of a large specific surface area, well-developed pores, good electrical conductivity, good electrolyte wetting, high chemical stability, and a wide potential window (Luo. 2020; Kim, 2021). The electrochemical properties of porous carbon electrode materials are a key factor affecting their energy storage properties.

Porous carbon materials are often used as anodes in batteries due to their good electrolyte wetting. The formation of uniform and elastic solid electrolyte interphase (SEI) or the use of tailored electrolytes can improve the stability of the SEI on the carbon surface, thereby increasing the safety and cyclability of the batteries (Fan, 2023; Gu, 2023). The porous carbon used as an electrode in a supercapacitor achieves a high specific capacitance and superior rate capability. Sun prepared nitrogen and sulfur co-doped carbon materials by chemical vapor deposition using magnesium hydroxide as a template, which had a large specific surface area (674 m^2^ g^-1^) and a porous interleaved network with a high level of heteroatom doping (Miao, 2019). As an anode material for Li-ion batteries, it shows a very high reversible capacity and excellent cycling stability.

### 3.3 Application of porous carbons in catalysis

The development of highly selective, catalytic, stable, green, and economically accessible catalysts is extremely important in industrial production. Carbon materials are often used as catalysts in CO_2_ electroreduction, oxygen reduction reactions (ORRs), and hydrogen evolution reactions (HERs) due to their abundance of sources and their excellent chemical properties.

Chen proposed an NH_3_ heat treatment strategy to completely remove pyrrole nitrogen and pyridine nitrogen dopants, and the prepared porous carbon material could efficiently electroreduce CO_2_, achieving a CO Faraday efficiency of 95.2% at a current density of −2.84 mA cm^-2^ (Dong et al., 2020). Saravanan treated peanut shells using a simple pyrolysis technique assisted by chemical activation and explored the HER properties of peanut shell-derived carbon nanosheets in acidic media (Saravanan, 2019). The nitrogen-doped carbon nanosheets with a high specific surface area and a large number of active sites showed excellent HER catalytic activity in aqueous electrolysis devices (Liu, 2020b). Lai synthesized two-dimensional porous disordered-layer carbon nanowebs with a large number of N-doped C defects using an aromatic ring as the carbon source and urea as the nitrogen source in a novel molecular design strategy (Lai, 2020). It was shown that carbon edge defects doped with graphitic N atoms could lead to materials exhibiting excellent ORR catalytic properties.

## 4 Discussions

Due to their high specific surface area, tunable physicochemical properties, low cost, and accessibility, porous carbon materials have shown a wide range of applications in areas such as catalysts, adsorbents, and energy storage. Previously, various methods were used to produce porous carbon materials with different pore structures, but most of the preparation was carried out in the laboratory with complex methods and processes, which made it difficult to meet the requirements of industrial preparation. Moreover, in order to regulate and optimize the structure of porous carbon materials at a more microscopic level and to point the way to the design and preparation of materials, further research on the mechanisms of action of carbon materials in their applications is needed. Thus, with the increasing energy shortage and environmental pollution, the further enhancement of the development of simple, clean, and cost-effective porous carbon materials could make a huge difference in a wider range of areas.
